# Inactivation of *Candida* Biofilms by Non-Thermal Plasma and Its Enhancement for Fungistatic Effect of Antifungal Drugs

**DOI:** 10.1371/journal.pone.0040629

**Published:** 2012-07-10

**Authors:** Yi Sun, Shuang Yu, Peng Sun, Haiyan Wu, Weidong Zhu, Wei Liu, Jue Zhang, Jing Fang, Ruoyu Li

**Affiliations:** 1 Department of Dermatology and Venereolgy, Peking University First Hospital, Research Center for Medical Mycology, Peking University, Beijing, China; 2 Academy for Advanced Interdisciplinary Studies, Peking University, Beijing, China; 3 College of Engineering, Peking University, Beijing, China; 4 Department of Applied Science and Technology, Saint Peter’s College, Jersey City, New Jersey, United States of America; Institute of Automation, Chinese Academy of Sciences, China

## Abstract

We investigated the antifungal effect of non-thermal plasma, as well as its combination with common antifungal drugs, against *Candida* biofilms. A direct current atmospheric pressure He/O_2_ (2%) plasma microjet (PMJ) was used to treat *Candida* biofilms in a 96-well plate. Inactivation efficacies of the biofilms were evaluated by XTT assay and counting colony forming units (CFUs). Morphological properties of the biofilms were evaluated by Scanning Electron Microscope (SEM). The sessile minimal inhibitory concentrations (SMICs) of fluconazole, amphotericin B, and caspofungin for the biofilms were also tested. Electron Spin Resonance (ESR) spectroscopy was used to detect the reactive oxygen species (ROS) generated directly and indirectly by PMJ. The *Candida* biofilms were completely inactivated after 1 min PMJ treatment, where severely deformed fungal elements were observed in SEM images. The SMICs of the tested antifungal drugs for the plasma-treated biofilms were decreased by 2–6 folds of dilution, compared to those of the untreated controls. ROS such as hydroxyl radical (^•^OH), superoxide anion radical (^•^O_2_^-^) and singlet molecular oxygen (^1^O_2_) were detected by ESR. We hence conclude that He/O_2_ (2%) plasma alone, as well as in combination with common antifungal drugs, is able to inactivate *Candida* biofilms rapidly. The generation of ROS is believed to be one of the underlying mechanisms for the fungicidal activity of plasma.

## Introduction

Candidiasis, caused by *Candida* species, is the most common fungal infection in humans [1], [2]. Beside invasive diseases including candidemia and candidiasis in deep-seated organ, mucocutaneous disorders such as oral candidiasis, vaginal and vulvovaginal candidiasis, have become a problem of significance in clinical practice [3], [4]. Although *Candida* species are the microorganism exhibiting planktonic unicellular form, they commonly show filamentous growth or complex multicellular structure in the infected tissues [5]. These structured microbial communities, known as biofilms, can attach to surfaces and encase within a matrix of exopolymeric materials [5], [6], and can form on various implanted medical devices such as vascular and urinary catheters, joint prostheses, cardiac valves, artificial vascular bypass devices, and those being topically used including contact lens and dentures [6]–[9]. The complex structure of biofilms makes them resistant to both host defense and commonly used antifungal drugs [6], [10], [11]. The yeast cells of *Candida* species, dropped constantly from the structured microbial communities, can spread and further cause antifungal treatment failure, devices failure or persistent infections [8], [12]. Currently, it is confirmed that the biofilm does contribute to the proliferation and the incidence rate of candidiasis [13]. Therefore, to develop novel approaches to inactivate candidal biofilm has great clinical practicability in treating candidiasis, especially those associated with biofilms.

Recently, as a novel therapeutic approach, atmospheric pressure non-thermal plasma has drawn much attention for its potential applications in clinical treatment, such as bacterial inactivation [14]–[16], blood coagulation [17], tooth whitening [18], [19], tumor treatment [20], as well as wound healing [21]. Compared with traditional therapeutic approaches, non-thermal plasma as a physical method could provide a more economic and effective way to manage a disease [22]. In addition, the gaseous form of plasma provides the possibility for the reactive species to penetrate into tissues of rough surfaces, cavities, fissures, and even down to spaces of micrometer scale. By endoscope, for example, the argon plasma has been widely used to treat the disorders even in deep-seated organ for years [23]–[25]. Therefore, it is also possible to combine this technique with minimally invasive surgery, endoscope, or antimicrobial chemotherapy for complete therapy. To date, several papers have reported the fungicidal effect of non-thermal plasma against *Candida albicans* [26], [27]. In addition, our group [28] have also found that non-thermal plasmas can effectively inactive the strains of *Candida* species including fluconazole-resistant *C. albicans*, *Candida glabrata* and *Candida krusei* in their planktonic form. And we also found that the antifungal susceptibility of various strains of *Candida* species to common antifungal drugs was enhanced after they were treated with plasma. These results indicated that non-thermal plasma could be a potential treatment method or supplementary treatment method for candidiasis. In the present study, we further investigated the fungicidal effect of non-thermal plasma on *Candida* biofilms, which are considered more difficult to inactive than their planktonic counterparts [29], [30], and the antifungal susceptibility of plasma-treated *Candida* biofilms to common antifungal drug.

## Results

### Plasma Inactivated *Candida* Biofilm Rapidly

**Figure 1:**
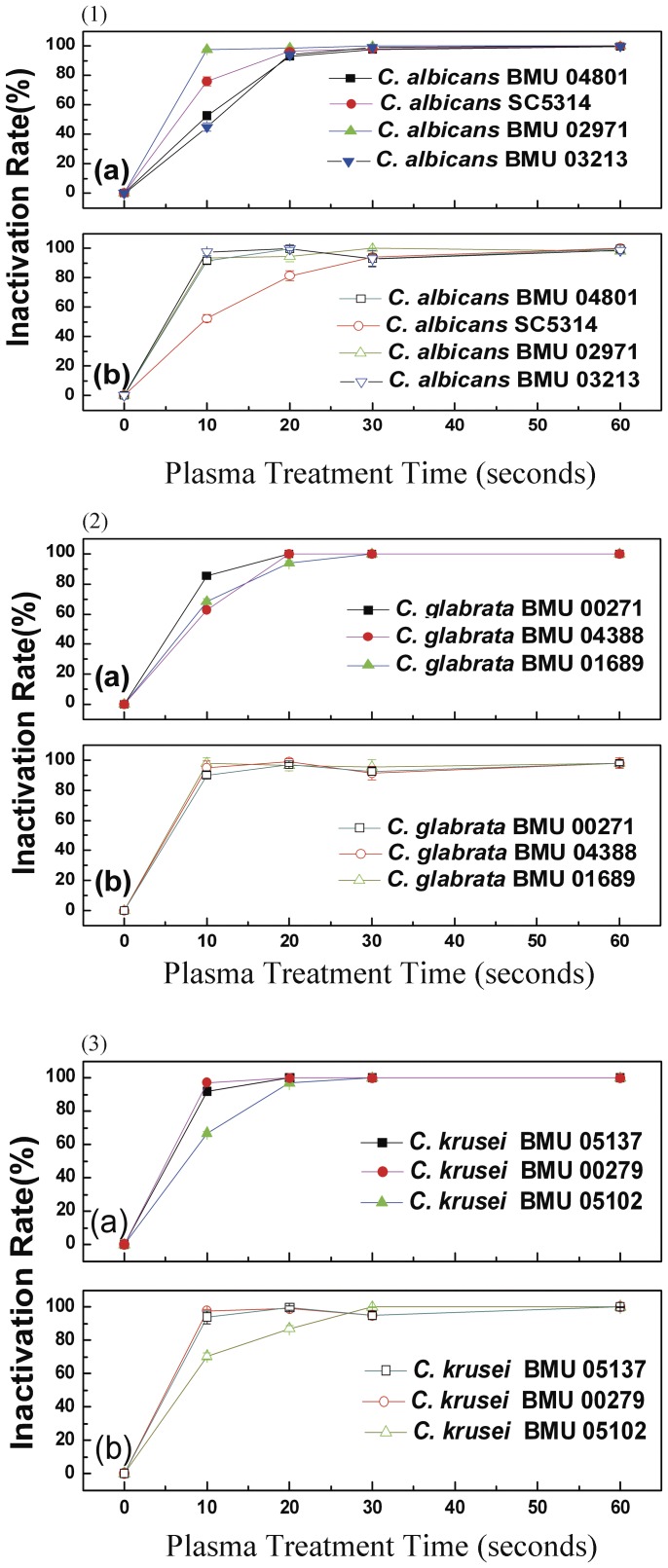
Evaluation of the inactivation rates of *Candida* biofilms treated with PMJ by CFU count (1-1a, 1-2a, 1-3a) and XTT assay (1-1b, 1-2b, 1-3b).

**Figure 2 pone-0040629-g002:**
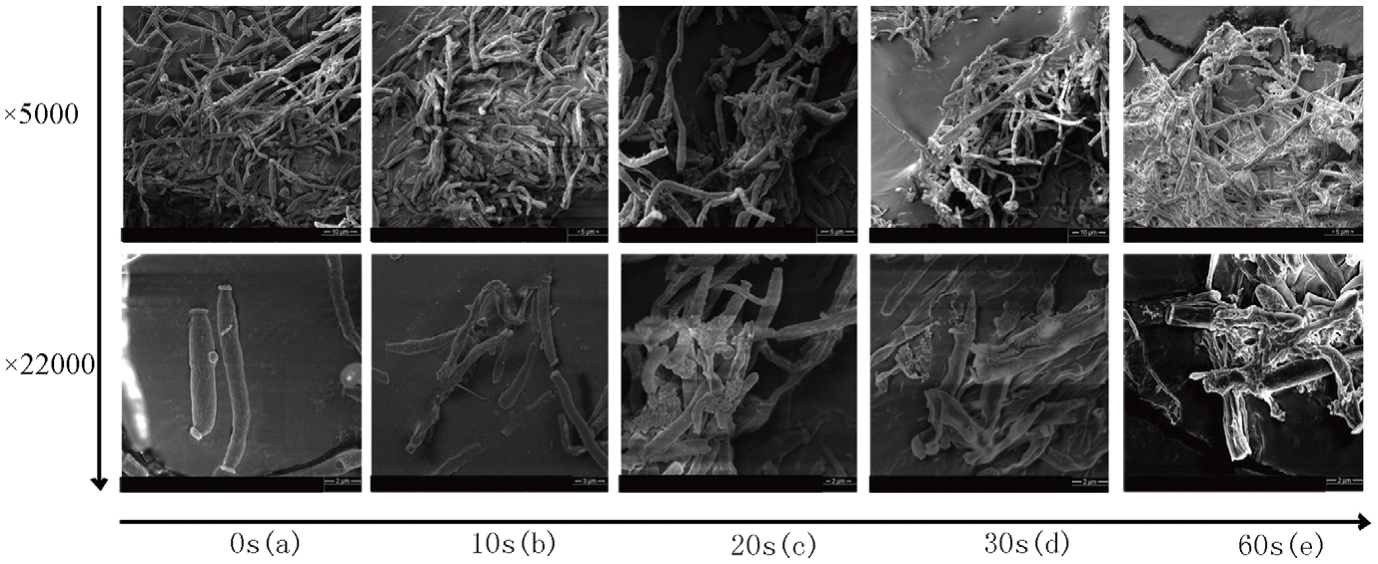
SEM results of *C. albicans* SC5314 biofilm before and after plasma treatment, the magnification scale were ×5000 and ×22000: (a), a negative control treated with He/O_2_ flow; (b), treated with PMJ for 10 s; (c), treated with PMJ for 20 s; (d), treated with PMJ for 30 s; (e), treated with PMJ for 60 s.

The inactivation rate of *Candida* biofilms (defined as (1-CFU_treated_/CFU_control_) ×100%) showed that 80% of the *Candida* biofilms was inactivated after 10 s PMJ treatment. The inactivation rate reached 90% after 30 s, and 100% after 60 s PMJ treatment (Fig. 1-1a, Fig. 1-2a, Fig. 1-3a).

XTT cell viability assay also showed 54%–96.6%, 81%–100%, and 91%–100% inactivation of *Candida* biofilms, after 10 s, 20 s, and 30 s of PMJ treatment, respectively (Fig. 1-1b, Fig. 1-2b, Fig. 1-3b), which were consistent with those observed by CFU counts, suggesting a rapid inactivation of *Candida* biofilms by PMJ treatment.

### Fungal Elements Embedded in *Candida* Biofilm were Severely Damaged After Treatment with Plasma

Healthy yeast cell and pseudohyphae with smooth surfaces were observed in biofilms treated with He/O_2_ gas flow (without PMJ), as shown in SEM images in Fig. 2-a. Samples treated with PMJ for 10 s showed rough surface of sessile cells and some fragmented cells (Fig. 2-b). When the treatment time was extended to 20 s, deformed and ruptured cells were observed (Fig. 2-c). Noticeably, yeast cells were cracked and pseudohyphae (crucial for biofilm formation) were ruptured after 30 s PMJ treatment (Fig. 2-d). When the treatment time was further extended to 60 s (Fig. 2-e), the biofilms lost their original morphological characteristics and degraded to clusters of cell fragments, which involved processes such as rupture, distortion and shrinking of the outer layer. This degradation may lead to the leakage of cell inclusion. The morphological changes of cell wall mentioned above were considered detrimental for the survival of the fungi, which were not seen in the negative control samples, where only He/O_2_ flow was introduced. In summary, highly structured community of *Candida* biofilm were gradually disrupted by PMJ. The biofilms formed by other strains showed similar results as *C. albicans* SC5314 displayed in SEM images.

### Free Radicals were Generated by He/O_2_ (2%) Non-thermal Plasma

**Figure 3:**
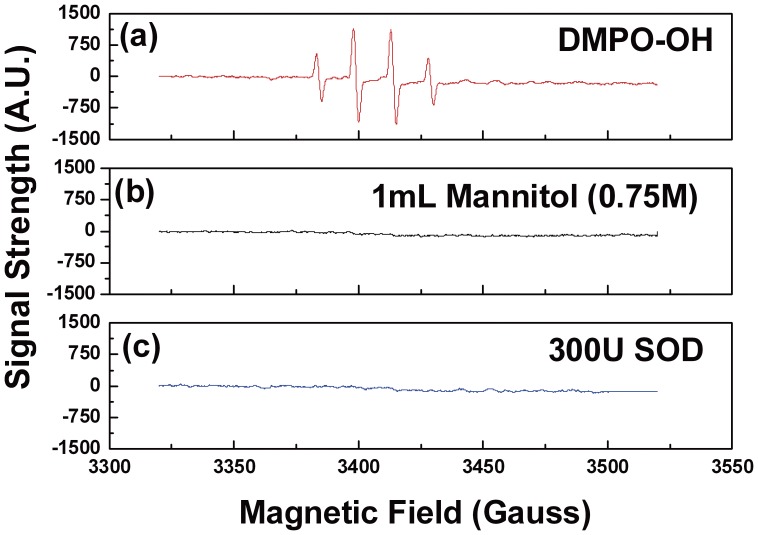
(a) The signal of DMPO-OH; (b) The influence of mannitol on the DMPO-OH signal; (c) The influence of SOD on the DMPO-OH signal.

^•^OH, with an oxidation potential of 2.8 eV, is the most reactive species among all ROS [31]. The lifetime of ^•^OH, however, is only nanoseconds in water. When spin-trapped by DMPO, the life time of DMPO-OH extends to minutes. As shown in Fig. 3-a, quartet DMPO-OH spectrum was observed, with hyperfine splitting constant a_N_ = a_H_ = 1.5 mT which conformed to the reported values [32]. When mannitol, a typical quencher of ^•^OH, was added, the DMPO-OH signal disappeared (Fig. 3-b), indicating the direct trapping of ^•^OH by DMPO.

O_2_^-•^ is both reductive and oxidative, and of low reactivity [33]. However, O_2_^-•^ can mediate the generation of ^•^OH via the well-known Haber-weiss reaction. When SOD (the sole enzyme for scavenging O_2_^-•^) was added, the DMPO-OH decreased rapidly (Fig. 3-c), indicating that ^•^OH was probably generated from O_2_^-•^ [34].

**Figure 4:**
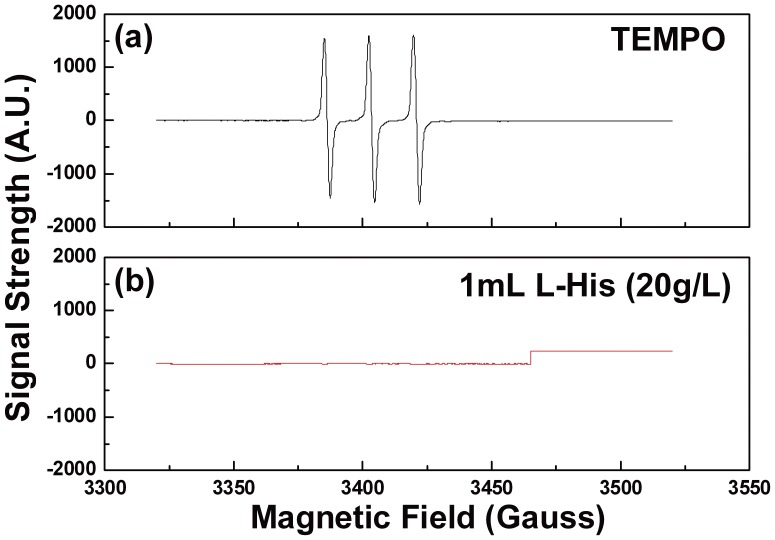
(a) The signal of TEMPO; (b) ^1^O_2_ existence verified by L-His .

^1^O_2_, which is also of high oxidative ability and can initiate lipid oxidation [35], might be more harmful compared to ^•^OH, considering its better diffusion in biological membranes and longer life-span (half-life in water: 3 µs) [36]. When TEMP was used as the spin trap, a strong triplet TEMPO signal was observed (Fig. 4-a). When L-His (scavenger of ^1^O_2_) was added into the system, the TEMPO signal decreased accordingly (Fig. 4-b). Therefore, ^1^O_2_ could also contribute to the inactivation process.

In summary, reactive oxygen species such as ^•^OH, O_2_^-•^and ^1^O_2_ were detected in the PMJ system. These ROS generated directly or indirectly by plasma are considered to have contributed partially to the inactivation of *Candida* biofilms.

### Enhancement of Fungistatic Effect of Common Antifungal Drugs on *Candida* Biofilm by Plasma Treatment

The minimal inhibitory concentrations (MICs) of amphotericin B (AMB), fluconazole (FLC), and caspofungin (CAP) against the planktonic cells of the mentioned 10 strains of *Candida* species are listed in Table 1.

**Table 1:** The SMIC50, 80 (µg/ml) and MIC (µg/ml) of FLC, AMB and CAP.

Isolate	Source	Species	Untreated(MIC)			Untreated (SMIC50/80)			10 s(SMIC50/80)			20 s(SMIC50/80)			30 s(SMIC50/80)		
			FLC	AMB	CAP	FLC*	AMB	CAP	FLC	AMB	CAP	FLC*	AMB*	CAP*	FLC*	AMB*	CAP*
BMU02971	Pharynx	*C.albicans*	≥64	2	1	≥256	0.5/4	1/2	≤1/8	≤0.125/0.5	≤0.015/0.5	≤1	≤0.125	≤0.015	≤1	≤0.125	≤0.015
BMU03213	Oral mucosa	*C.albicans*	1	0.5	0.5	≥256	0.25/0.5	0.25/0.5	≤1/4	≤0.125/0.25	≤0.015/0.5	≤1	≤0.125	≤0.015	≤1	≤0.125	≤0.015
BMU04801	Oral mucosa	*C.albicans*	2	2	0.5	≥256	1/4	0.5/1	8/32	0.25/0.25	0.06/0.5	≤1	≤0.125	≤0.015	≤1	≤0.125	≤0.015
SC5314	Blood	*C.albicans*	2	1	0.5	≥256	1/2	0.25/0.5	16/64	0.25/0.5	0.06/0.25	≤1	≤0.125	≤0.015	≤1	≤0.125	≤0.015
BMU00279	Sputum	*C.krusei*	≥64	1	1	≥256	1/1	1/1	≤1/4	≤0.125/0.25	≤0.015/0.5	≤1	≤0.125	≤0.015	≤1	≤0.125	≤0.015
BMU05102	Oral mucosa	*C. krusei*	≥64	1	0. 5	≥256	0.5/1	0.25/0.5	≤1/8	≤0.125/0.25	≤0.015/0.25	≤1	≤0.125	≤0.015	≤1	≤0.125	≤0.015
BMU05137	Oral mucosa	*C. krusei*	≥64	1	2	≥256	2/4	0.5/1	2/16	≤0.125/0.25	≤0.015/0.03	≤1	≤0.125	≤0.015	≤1	≤0.125	≤0.015
BMU00271	Blood	*C.glabrata*	≥64	1	0.5	≥256	0.5/1	0.25/0.5	≤1/4	≤0.125/0.25	≤0.015/0.25	≤1	≤0.125	≤0.015	≤1	≤0.125	≤0.015
BMU01689	Knee	*C.glabrata*	32	2	1	≥256	0.5/2	0.25/1	2/8	≤0.125/0.25	≤0.015/0.25	≤1	≤0.125	≤0.015	≤1	≤0.125	≤0.015
BMU04388	Intraperitoneal fluid	*C.glabrata*	32	1	0.5	≥256	0.5/1	1/2	2/4	≤0.125/0.5	0.5/1	≤1	≤0.125	≤0.015	≤1	≤0.125	≤0.015

MIC, minimal inhibitory concentration, used for evaluating the antifungal activity of antifungal drugs against planktonic cell of *Candida* species; SMIC, sessile minimum inhibitory concentration, used for evaluating the antifungal activity of antifungal drugs against *Candida* biofilms.

* SMIC50 values were equal to SMIC80 values.

The sessile minimum inhibitory concentrations (SMICs) of FLC, AMB, and CAP (Table 1) of the *Candida* biofilms treated with PMJ for 10 s, 20 s and 30 s, respectively, showed a significant reduction, compared with those of the untreated groups.

After being treated with plasma for 20 s or 30 s, small part of the *Candida* species cells in the *Candida* biofilms survived while most of plasma-treated biofilms were inactivated, as shown by the CFU and XTT results after being incubated in RPMI1640 medium for 24 h at 37°C.

The antifungal activity of the common antifungal drugs against *Candida* biofilms treated with PMJ for 10 s showed that the SMIC50 ranges of FLC, AMB and CAP were 1–16 µg/ml, 0.125–0.25 µg/ml, and 0.015–0.5 µg/ml, respectively, and the SMIC80 ranges of these drugs were 4–64 µg/ml, 0.125–0.5 µg/ml and 0.03–0.5 µg/ml, respectively. The SMIC50****s and SMIC80****s of FLC, AMB and CAP against *Candida* biofilms treated with PMJ for 20 s and 30 s were ≤1 µg/ml, ≤0.125 µg/ml and ≤0.015 µg/ml (Table 1), respectively. Since the *Candida* biofilms were completely inactivated after 60 s, the antifungal activity of the common antifungal drugs was not evaluated for those groups.

## Discussion

The increased using of implanted devices has facilitated the formation of *Candida* biofilms, which further accelerates infections caused by *Candida* species. In addition, *Candida* biofilm is resistant to both host defense and commonly used antifungal drugs [7], [8], [12], [13], [37]. Therefore, a series of studies on *Candida* biofilm development, structure properties, especially its susceptibility to commonly used antifungal drugs have been carried out using various *in vitro* models [29], [38], [39].

We found that the SMIC80 ranges of AMB, FLC, and CAP against the biofilms, induced *in vitro* by 10 strains of *Candida* species on 96-well plate, were 0.5–2 µg/ml, ≥256 µg/ml, and 0.5–2 µg/ml, respectively (as well as SMIC50 ranges were shown in Table 1), while the MIC ranges of these antifungals, determined by using Clinical and Laboratory Standard Institute (CLSI) broth microdilution method [39], against the planktonic cells of the mentioned 10 strains of *Candida* species were 0.5–2 µg/ml, 1-≥64 µg/ml, and 0.5–2 µg/ml, respectively (Table 1). These results, which showed the susceptibility of biofilms to common antifungal drugs was lower than that of their planktonic counterparts, were consistent with previous reports [11], [40], indicating poor therapeutic effect on these biofilms.

Based on our previous observation [28], non-thermal plasma could effectively inactivate the planktonic cells of *Candida* species and enhance the susceptibility of *Candida* species to common antifungal drugs. In this experiment, we treated the biofilms of 10 strains of *Candida* species with non-thermal plasma. Via CFU counting, we found that 80% of *Candida* biofilms were inactivated after 10 s of treatment, a 90% inactivation after 30 s of treatment, and a 100% inactivation after 60 s of treatment (Fig. 1). XTT cell viability assay showed that 54%–96.6% of biofilms were inactivated after 10 s of treatment, and the inactivation rates observed after 30 s and 60 s of treatment are comparable with those obtained via CFU counting (Fig. 1). Furthermore, fungal elements including mycelia within the biofilms induced by the strain of *C. albicans* SC5314 were severely damaged. And the severity of mycelium damage increased with the plasma treatment duration (Fig. 2). All these results prompted strong and rapid inactivation capability of non-thermal plasma to biofilms of various strains of *Candida* species, whether it being resistant to common antifungal drugs or not. Without a doubt, these observations provided the basis for developing novel approaches in treating candidiasis with biofilm.

It was shown in our previous study [28] that He/O_2_ (2%) non-thermal plasma inactivated the planktonic cells of *Candida* species in both air and water via partially reactive oxygen species (ROS). In a different study [41], we have also observed that the oxidative stress pathway of eukaryotic cells *Saccharomyces cerevisiae* can lead to increased sensitivity to plasma treatment. More recently [42], it was found that the addition of SOD, D-Man and L-His as scavengers of ROS resulted in significantly decreased ability of the plasma for the inactivation of *S. cerevisiae*. It is likely that ROS generated by plasma contribute partially to the effective inactivation of *Candida* biofilms, too. The existence of ROS such as ^•^OH, O_2_^-•^ and ^1^O_2_ in the He/O_2_(2%) non-thermal plasma – liquid system was confirmed by ESR spectroscopy. These reactive oxygen species were proven to cause lipids and proteins of cytomembrane to suffer from oxidative damage which leads to impediment of ion transition. Furthermore, the oxidative molecules could combine with DNA to induce additional cell damages [43], [44].

Antifungal drugs, despite of poor outcome, are now commonly used for treating *Candida* biofilms besides the choice of removing implanted devices [8], [13]. In our study, it was found that the SMICs of commonly used antifungal drugs for the *Candida* biofilms treated with plasma for very short time (10–30 s) were reduced significantly (Table 1). And it was also found the SMICs of FLC for the plasma-treated biofilms induced by strains of *C. albicans*, *C. glabrata* and *C. krusei*, being resistant or susceptible dose-dependent to FLC, were similar to that for the plasma-treated biofilm induced by the strain of *C. albicans* SC5314 (Table 1). These observations prompted that He/O_2_ (2%) non-thermal plasma could become a novel supplementary approach to antifungal drug therapy for candidiasis related to biofilm, which could be induced by the strains being either resistant or not to the administered drugs.

However, other issues concerning the safety of operation and reactive species other than the detected ROS, as well as its mode of operation under different circumstances need to be further investigated, with the aim to provide the basis for developing novel antifungal approaches or supplementary approaches in treating candidiasis with biofilm.

## Materials and Methods

### Biofilm Preparation

Ten strains of *Candida* species, including 4 strains of *C. albicans*, 3 strains of *C. glabrata*, and 3 strains of *C. krusei*, used in this study were isolated and stored in our laboratory as shown in Table 1.

After each strain was grown on potato dextrose agar (PDA) at 35°C for 3 days to ensure the viability and purity, the biofilm models were established as described previously [40] with minor modification: a loopful of *Candida* cells was inoculated in 20 ml yeast peptone dextrose liquid medium and incubated overnight in a shaker (150 rpm) at 30°C. Cells were harvested from the overnight-grown liquid cultures by centrifugation (3000 g×5 min at 4°C), and were then washed by ice-cold sterile PBS. RPMI-1640 broth medium was used to re-suspend the pellet and adjust the final density to 1.0×10^6^ cells/ml for all strains. One hundred microliter of prepared suspension was pipetted into selected wells of 96-well microtiter plates and every replication was separated by an empty well. After incubating statically for 24 hours at 37°C, the biofilm was formed. The medium was aspirated from the wells which were then washed with sterile water three times to remove planktonic cells.

### Plasma Treatment

The plasma device used in our studies is comprised of two copper tubes separated by a ceramic tube and driven by a direct current high voltage power supply. Details of the plasma device and the electrical circuitry can be found in the references [16], [45]. Premixed helium and oxygen (volume ratio: 98% He and 2% O_2_, referred to He/O_2_ from here on) was used as the working gas at a flow rate of 2.5 slm. Biofilms were treated by non-thermal plasma micro-jet (PMJ) in a 96-well plate for 10 seconds, 20 seconds, 30 seconds and 60 seconds, respectively. The procedure was repeated for at least 30 wells at each time point.

### CFU Count and XTT Assay

After PMJ treatment, 100 µl of sterile water was added into the well and washed vigorously in order to re-suspend the biofilm cells thoroughly. Then, suspension was diluted 1000 times with sterile water and 100 µl of it was then pippeted out and spread evenly by a sterile plastic transferring loop on SDA. The CFU count was performed via a counting program developed in house after 24 h incubation on SDA at 35°C. The inactivation rate was calculated as (1-CFU_treated_/CFU_control_) ×100%.

In XTT cell viability assay, a mixture of 100 µl RPMI1640 broth medium, 100 µl 2,3-bis (2-methoxy-4-nitro-5-sulfophenyl) [phenyl-amino)car-bonyl] -2H-tetrazolium hydroxide (XTT, 1 mg/ml, Sigma-Aldrich Co., St. Louis, USA) and menadione (10 mM, Sigma-Aldrich Co., St. Louis, USA) was added into wells treated by PMJ. Covered with aluminum foil, the plates were incubated at 37°C for 2 h. Using a multichannel pipette, 80 µl of the resulting colored supernatant was removed and transferred into the wells of new microtiter plates which would be measured by a microtiter plate reader (Bio-rad680). The optical density (OD) of the supernatant in each well was determined by measuring the absorbance at 490 nm. Every group contained negative and positive controls. The inactivation rate was calculated as (1-OD_treated_/OD_control_) ×100%.

### Scanning Electron Microscopy (SEM)

After PMJ treatment, biofilm formed by strain *C. albicans* SC5314 was fixed with 2.5% glutaraldehyde overnight and was then dehydrated sequentially in ethanol (30%, 50%, 70%, 80%, 90%, 100%). The samples were gold-paladium coated and observed by a SEM (Quanta 200FEG and NOVA NANOSEM 430) to evaluate the morphological properties of *Candida* biofilms. Biofilm of *C. albicans* SC5314 without PMJ but with He/O2 gas flow treatment was used as control.

### Electron Spin Resonance (ESR) Spectroscopy

Electron Spin Resonance (ESR) spectroscopy, which has been widely used for the detection of free radicals [33], [46], was applied to evaluate the reactive species generated by PMJ. In principal, ESR is a direct method for detecting species which possesses an unpaired electron, whose spin states are split in an external magnetic field. Upon application of a magnetic field of the resonance frequency between the two states, a transition is induced, which is signaled by an absorption peak on the spectrum of the magnetic field. As most of reactive species have rather short life-span and are therefore difficult to be detected in ESR spectrum, spin trapping was applied to facilitate the detection by reacting short-lived radicals with a spin trap reagents. As a result, persistent aminoxyl spin adduct radicals are produced, which have longer life-span and are thus more favorable in ESR detection. In this experiment, DMPO (5,5-dimethyl-1-pyrroline-N-oxid, Sigma Aldrich Co., Ltd) and TEMP (2,2,6,6-Tetramethylpiperidine, Sigma Aldrich Co., Ltd) were used as the spin trap reagents for hydroxyl radical (^•^OH) and singlet oxygen (^1^O_2_) respectively, resulting in spin adducts DMPO-OH and TEMPO. DMPO-OH is characterized by a 1∶2:2∶1 quartet spectrum, while TEMPO shows a 1∶1:1 triplet spectrum. Detailed information can be found in references [19], [41].

20 µl DMPO (0.8 mol/L) was added into 1 mL distilled water and was treated by plasma for 20 seconds (PMJ was sustained 1 cm above water). In comparison, Superoxide Dismutase (SOD, Sigma Aldrich Co., Ltd), the sole enzyme for scavenging O_2_^-•^, was added into 1 ml water before the addition of DMPO, to clarify the relationship between the DMPO-OH signal and superoxide anion radical (O_2_^-•^). Similarly, 20 µl TEMP (99.9%) was added into 1 mL distilled water, and then treated by air PMJ for 20 seconds (PMJ was also sustained 1 cm above water). L-Histidine (L-His, Shanghai Secondary Military Science Academy), commonly used for scavenging ^1^O_2_, was added into water before adding TEMP, to validate the contribution of ^1^O_2_ to the signal.

After each PMJ treatment, about 40 µl samples was imbibed by a capillary tube and sent to ESR resonance chamber. Measurements of the ESR signals were carried out on an ER-200D-SRC ESR spectrometer (Bruker Ltd, Germany) operated at room temperature under the following conditions: central magnetic field, 3420.00 Gauss; sweep width, 200.0 Gauss; frequency, 9.54 GHz; modulation frequency, 100 kHz and power 20 mW.

### Antifungal Susceptibility Test

The antifungal susceptibility test was performed according to the method as described previously [40]. The concentration range of FLC (Fuyang Genebest Chemical Industry Co. Ltd, China) was 1–256 µg/ml, CAP (Merck, NJ, USA) was 0.015–2 µg/ml, and AMB (Sigma-Aldrich Co., St. Louis, USA) was 0.125–16 µg/ml. One hundred microliters of the serially diluted drug solutions were added into wells containing plasma-treated biofilms. And the untreated biofilms were used as growth controls. After incubated for 24 h at 37°C, the metabolic activities of plasma-treated biofilms were assayed by XTT prepared as described above. From the resulting colorimetric readings, after subtracting the corresponding values of negative controls, the SMICs for the *Candida* biofilm was calculated: SMIC50 and SMIC80 were the drug concentrations at which a 50% or 80% decrease in absorbance was detected in comparison with the positive control biofilms. And antifungal susceptibility of planktonic cells of these *Candida* strains was assayed by using CLSI broth microdilution method M27-A3 [39]. All experiment procedures were performed three times for statistical analysis.

## References

[1] Concia E, Azzini AM, Conti M (2009). Epidemiology, incidence and risk factors for invasive candidiasis in high-risk patients.. Drugs.

[2] Arendrup MC (2010). Epidemiology of invasive candidiasis.. Curr Opin Crit Care.

[3] Rowen JL (2003). Mucocutaneous candidiasis.. Semin Perinatol.

[4] Havlickova B, Czaika VA, Friedrich M (2008). Epidemiological trends in skin mycoses worldwide.. Mycoses.

[5] Hasan F, Xess I, Wang X, Jain N, Fries BC (2009). Biofilm formation in clinical *Candida* isolates and its association with virulence.. Microbes Infect.

[6] D’Enfert C (2006). Biofilms and their role in the resistance of pathogenic *Candida* to antifungal agents.. Curr Drug Targets.

[7] Ramage G, Tomsett K, Wickes BL, Lopez-Ribot JL, Redding SW (2004). Denture stomatitis: a role for *Candida* biofilms.. Oral Surg Oral Med Oral Pathol Oral Radiol Endod.

[8] Ramage G, Martinez JP, Lopez-Ribot JL (2006). *Candida* biofilms on implanted biomaterials: a clinically significant problem.. FEMS Yeast Res.

[9] Bryers JD, Ratner BD (2004). Bioinspired implant materials befuddle bacteria.. Asm News.

[10] Ramage G, Wickes BL, Lopez-Ribot JL (2001). Biofilms of *Candida albicans* and their associated resistance to antifungal agents.. Am Clin Lab.

[11] Mukherjee PK, Chandra J. Candida biofilm resistance (2004). Drug Resist Updat.

[12] Cauda R (2009). Candidaemia in patients with an inserted medical device.. Drugs.

[13] Kojic EM, Darouiche RO (2004). *Candida* infections of medical devices.. Clin Microbiol.

[14] Laroussi M (2002). Nonthermal decontamination of biological media by atmospheric-pressure plasmas: Review, analysis, and prospects.. IEEE Trans Plasma Sci.

[15] Kim SJ, Chung TH, Bae SH, Leem SH (2009). Bacterial inactivation using atmospheric pressure single pin electrode microplasma jet with a ground ring.. Appl Phys Lett.

[16] Feng H, Sun P, Chai Y, Tong G, Zhang J (2009). The Interaction of a direct-current cold atmospheric-pressure air plasma with bacteria.. Ieee T Plasma Sci.

[17] Fridman G, Peddinghaus M, Ayan H, Fridman A, Balasubramanian M (2006). Blood coagulation and living tissue sterilization by floating-electrode dielectric barrier discharge in air.. Plasma Chem Plasma Process.

[18] Lee HW, Kim GJ, Kim JM, Park JK, Lee JK (2009). Tooth bleaching with nonthermal atmospheric pressure plasma.. J Endod.

[19] Sun P, Pan J, Tian Y, Bai N, Wu H (2010). Tooth-whitening with hydrogen peroxide assisted by a direct current, cold, atmospheric-pressure air plasma microjet.. Ieee T Plasma Sci.

[20] Vandamme M, Robert E, Pesnel S, Barbosa E, Dozias S (2010). Antitumor effect of plasma treatment on U87 glioma xenografts: preliminary results.. Plasma Process Polym.

[21] Nosenko T, Shimizu T, Morfill GE (2009). Designing plasmas for chronic wound disinfection.. New J Phys.

[22] Heinlin J, Isbary G, Stolz W, Morfill G, Landthaler M (2011). Plasma applications in medicine with a special focus on dermatology.. J Eur Acad Dermatol Venereol.

[23] Schulz H, Miehlke S, Antos D, Schentke KU, Vieth M (2000). Ablation of Barrett’s epithelium by endoscopic argon plasma coagulation in combination with high-dose omeprazole.. Gastroint Endos.

[24] Malick KJ (2006). Clinical applications of argon plasma coagulation in endoscopy.. Gastroenterol Nurs.

[25] Raiser J, Zenker M (2006). Argon plasma coagulation for open surgical and endoscopic applications: state of the art. J. Phys. D: Appl. Phys..

[26] Rupf S, Lehmann A, Hannig M, Schafer B, Schubert A (2010). Killing of adherent oral microbes by a non-thermal atmospheric plasma jet.. J Med Microbiol.

[27] Morfill GE, Shimizu T, Steffes B, Schmidt H (2009). Nosocomial infections–a new approach towards preventive medicine using plasmas.. New J Phys.

[28] Sun P, Sun Y, Zhou H, Wu H, Zhu W (2011). Atmospheric pressure cold plasma as an antifungal therapy.. Appl Phys Lett.

[29] Tobudic S, Kratzer C, Lassnigg A, Graninger W, Presterl E (2010). *In vitro* activity of antifungal combinations against *Candida albicans* biofilms.. J Antimicrob Chemother.

[30] Seneviratne CJ, Jin LJ, Samaranayake YH, Samaranayake LP (2008). Cell density and cell aging as factors modulating antifungal resistance of *Candida albicans* biofilms.. Antimicrob Agents Chemother.

[31] Venkatadri R, Peters RW (1993). Chemical oxidation technologies: ultraviolet light/hydrogen peroxide, fenton’s reagent, and titanium dioxide-assisted photocatalysis.. Hazatdous Waste & Hazrdous Materials.

[32] Buettner GR (1987). Spin trapping: ESR parameters of spin adducts.. Free Radic Biol Med.

[33] Koppenol WH (2001). The Haber-Weiss cycle–70 years later.. Redox Rep.

[34] Sawyer DT, Valentine JS (1981). How super is superoxide?. Acc.Chem.Res.

[35] Frankel EN (1980). Lipid oxidation.. Prog Lipid Res.

[36] Gorman AA, Rodgers MAJ (1978). Lifetime and reactivity of singlet oxygen in an aqueous micellar system: a pulsed nitrogen laser study.. Chem Phys Lett.

[37] Dasgupta MK (2002). Biofilms and infection in dialysis patients.. Semin Dial.

[38] Baillie GS, Douglas LJ (2000). Matrix polymers of *Candida* biofilms and their possible role in biofilm resistance to antifungal agents.. J Antimicrob Chemother.

[39] Clinical and Laboratory Standards Institute (2008). Reference method for broth dilution antifungal susceptibility testing of yeasts; approved standard –– M27-A3.. CLSI, Wayne, PA, USA.

[40] Pierce CG, Uppuluri P, Tristan AR, Jr Wormley FL, Mowat E (2008). A simple and reproducible 96-well plate-based method for the formation of fungal biofilms and its application to antifungal susceptibility testing.. Nat Protoc.

[41] Feng H, Wang R, Sun P, Wu H, Liu Q (2010). A study of eukaryotic response mechanisms to atmospheric pressure cold plasma by using *Saccharomyces cerevisiae* single gene mutants.. Appl Phys Lett.

[42] Ma R, Feng H, Li F, Liang Y, Zhang Q (2012). An evaluation of anti-oxidative protection for cells against atmospheric pressure cold plasma treatment.. Appl Phys Lett.

[43] Davies MJ (2003). Singlet oxygen-mediated damage to proteins and its consequences.. Biochem Biophys Res Commun.

[44] Buonocore G, Perrone S, Tataranno ML (2010). Oxygen toxicity: chemistry and biology of reactive oxygen species.. Semin Fetal Neonatal Med.

[45] Liu F, Sun P, Bai N, TianYe, Zhou H (2010). Inactivation of bacteria in an aqueous environment by a direct-current, cold-atmospheric-pressure air plasma microjet.. Plasma Processes and Polymers.

[46] Halliwell B, Whiteman M (2004). Measuring reactive species and oxidative damage *in vivo* and in cell culture: how should you do it and what do the results mean?. Br J Pharmacol.

